# Supplementary material for: The dynamic properties of a nuclear coactivator binding domain are evolutionarily conserved
Source: Commun Biol. 2022 Mar 30;5:286. doi: 10.1038/s42003-022-03217-y (PMC8967867; doi:10.1038/s42003-022-03217-y)
Supplement: Supplementary file 2 — Supplementary information [file 42003_2022_3217_MOESM2_ESM.pdf]

Supplementary information

The dynamic properties of a nuclear coactivator binding domain are evolutionarily conserved

Elin Karlsson[1], Frieda A. Sorgenfrei[1,†], Eva Andersson[1], Jakob Dogan[1], Per Jemth[1,*], and Celestine N. Chi[1,2,*]

[1]Department of Medical Biochemistry and Microbiology, Uppsala University, BMC Box 582, SE-75123 Uppsala, Sweden.

[2]Department of Pharmaceutical Biosciences, Uppsala University, BMC Box 582, SE-75123 Uppsala, Sweden

[†]Present address: acib GmbH, Krenngasse 37, 8010 Graz c/o University of Graz, Institute of Chemistry, NAWI Graz, BioTechMed Graz, Heinrichstrasse 28, 8010 Graz, Austria

*Correspondence to

Celestine Chi, Celestine.Chi@imbim.uu.se

Per Jemth, Per.Jemth@imbim.uu.se

Supplementary figures

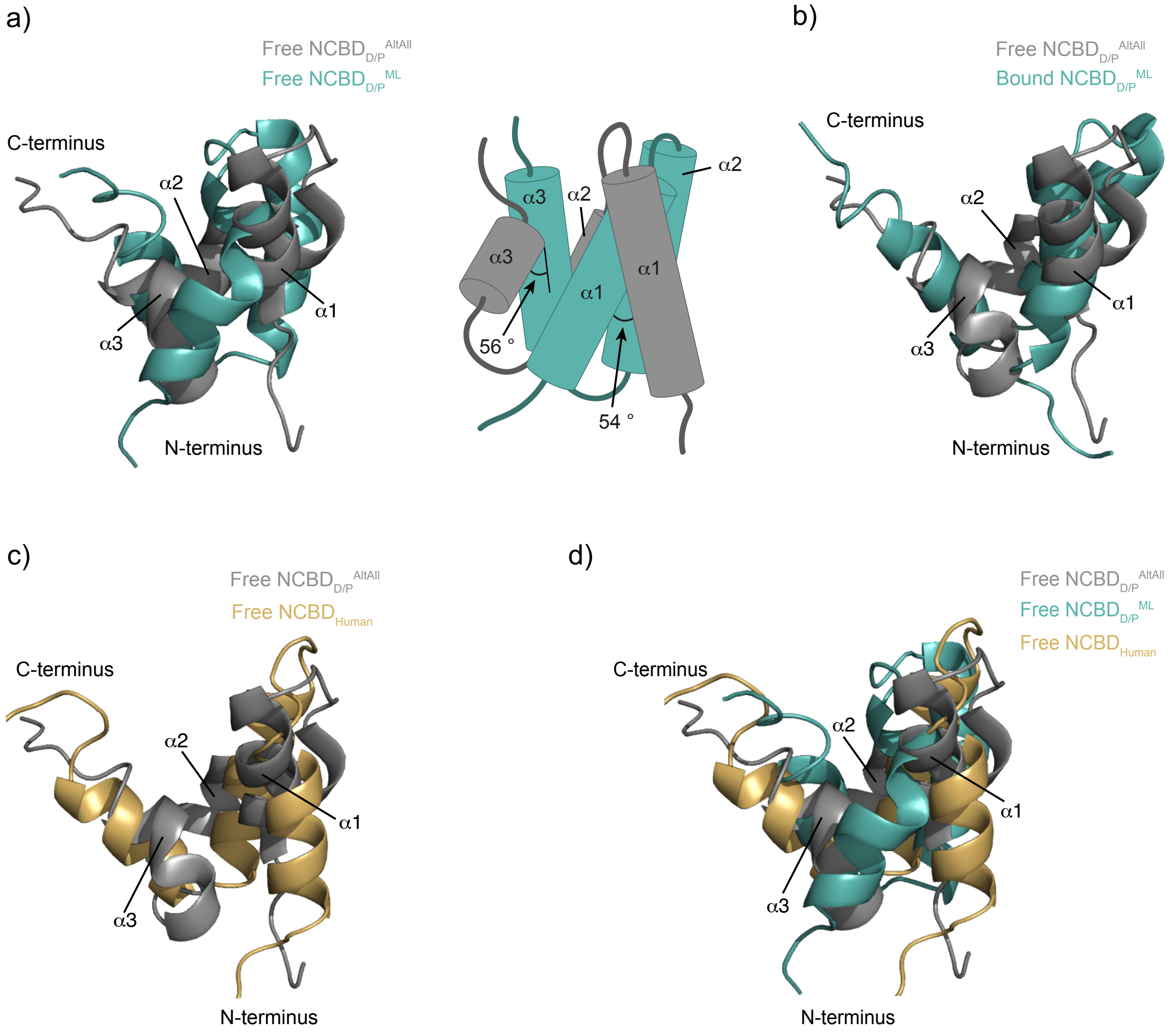

Figure S1. Overlay of the structures of NCBD$_{D/P}^{ML}$, NCBD$_{D/P}^{AltAll}$ and NCBD$_{Human}$. (a) Overlay of NCBD$_{D/P}^{AltAll}$ (7OSW) and NCBD$_{D/P}^{ML}$ (7OSR). Tilt angles of helices are indicated in the schematic figure and explained in Table S1. (b) Overlay of NCBD$_{D/P}^{AltAll}$ (7OSW), and the previously determined CID$_{1R}^{ML}$-bound NCBD$_{D/P}^{ML}$ (6ES5). (c) Overlay of NCBD$_{D/P}^{AltAll}$ (7OSW), and the previously determined NCBD$_{Human}$ (2KKJ). (d). An overlay of the structures determined from NMR for NCBD$_{D/P}^{AltAll}$ (7OSW), NCBD$_{D/P}^{ML}$(pdb code: 7OSR), and the previously determined NCBD$_{Human}$ (2KKJ).

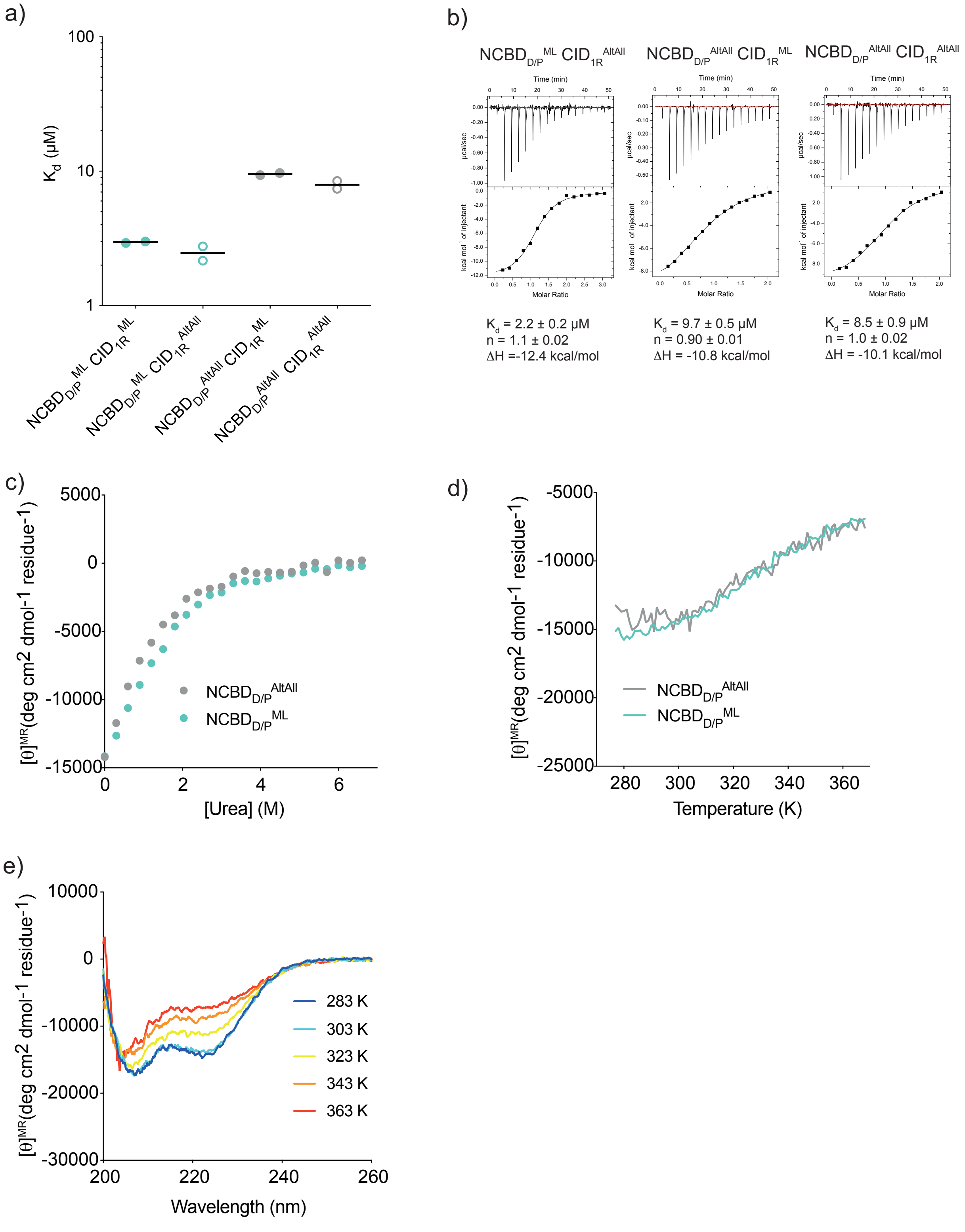

Figure S2. The biophysical properties of NCBD$_{D/P}$^{ML} are robust to errors in the sequence. a) Binding affinities of NCBD$_{D/P}$^{ML} or NCBD$_{D/P}$^{AltAlt} and CID$_{1R}$^{ML} or CID$_{1R}$^{AltAll} measured with ITC. The buffer solution was 20 mM sodium phosphate pH 7.4, 150 mM NaCl. The two data points for the NCBD$_{D/P}$^{ML}: CID$_{1R}$^{ML} complex are from Hultqvist *et al.* (2017) *eLife* 6, e16059 and Karlsson *et al.* (2020) *J. Biol. Chem.* 295, 17698. (b) Representative ITC thermograms for NCBD:CID complexes. The data were fitted to a two-

state model, which yielded estimates of K_d, ΔH and the number of binding sites n. The fitted parameters along with the estimated errors are shown below each thermogram. (c) Stability of $NCBD_{D/P}^{AltAll}$ measured by urea denaturation in 50 mM potassium formate (pH 3.0), 150 mM NaCl. The CD signal at 222 nm was monitored and the data was fitted to a two-state function. A qualitative comparison with $NCBD_{D/P}^{ML}$ indicates that the stability of the two variants is similar. See Supplementary Fig. S3 and Table S2 for a quantitative comparison. (d-e) Temperature stability for $NCBD_{D/P}^{AltAll}$. The CD signal at 222 nm was monitored (d) and spectra were taken at temperatures between 283-363 K (e) in 50 mM potassium formate (pH 3.0), 150 mM NaCl.

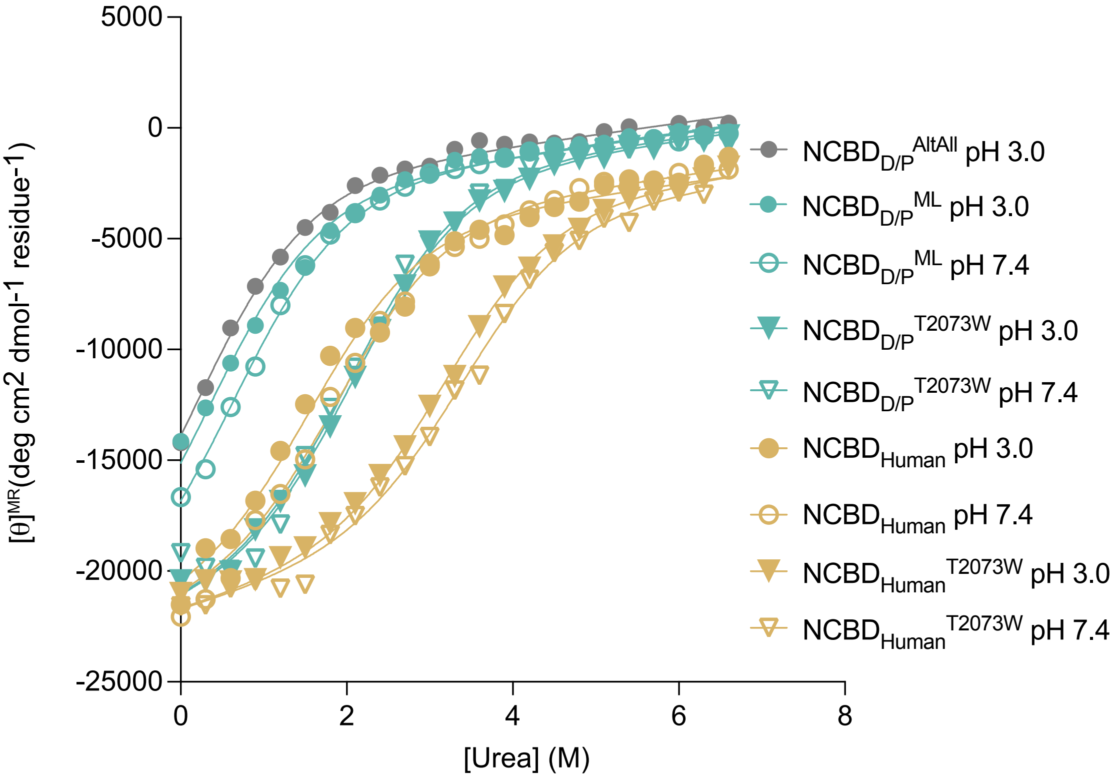

Figure S3. Equilibrium urea denaturation experiments. All data sets were fitted simultaneously to a two-state model in GraphPad Prism with shared parameters for the native state molar ellipticity, the denatured and native baseline slopes and the $m_{D\text{-}N}$ value. Experiments were performed in either 50 mM potassium formate buffer (pH 3.0), 150 mM NaCl or 20 mM sodium phosphate buffer (pH 7.4), 150 mM NaCl. The shared $m_{D\text{-}N}$ value was fitted as 0.82 ± 0.03 kcal mol^{-1}M^{-1}. Values for $\Delta G_{D\text{-}N}$ and [Urea]$_{50\%}$ are shown in Table S2.

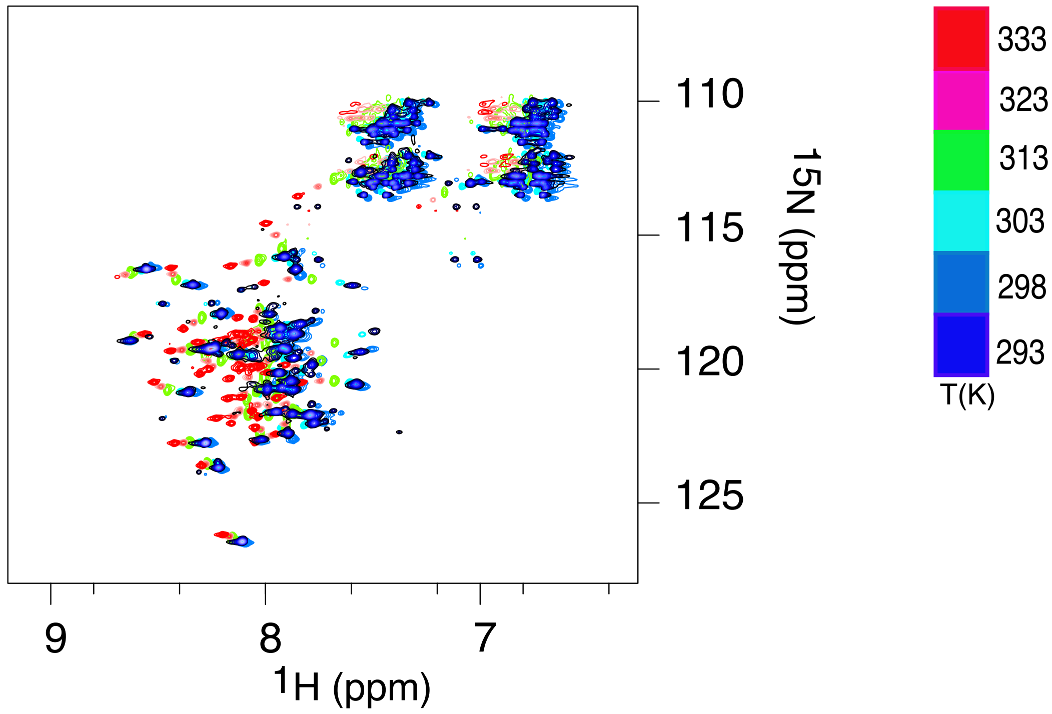

Figure S4. ^{15}N-^{1}H HSQC spectra of NCBD$_{D/P}^{ML}$ at different temperatures. The experiments were performed from 293 K (blue) to 333 K (red) at pH 2.4.

Supplementary Tables

Table S1. Helix tilt angles and SASA. Tilt angles for the NCBD variants were measured using structural alignments in PyMol.[1] Solvent accessible surface area (SASA) was calculated by ProtSA[2] using the lowest energy structures and a solvent radius of 1.4 Å. Residues included were 2062-2109 (numbering according to human CBP), see Fig. 1 in the main text.

NCBD variant	pH for structure	α1 tilt angle versus $NCBD_{D/P}^{ML}$	α3 tilt angle versus $NCBD_{D/P}^{ML}$	SASA folded (Å^2)	ΔSASA (unfolded-folded) (Å^2)
$NCBD_{D/P}^{ML}$ 7OSR	2.4	0	0	4721	1264
$NCBD_{D/P}^{AltAll}$ 7OSW	6.8	54°	56°	4260	1795
$NCBD_{Human}$ 2KKJ	6.5	63°	5.8°	4164	1825

[1]We measured the angles between the first helix α1 of $NCBD_{D/P}^{ML}$ and $NCBD_{Human}$ using the following Cα carbons: Gln2069 $NCBD_{D/P}^{ML}$ and $NCBD_{Human}$, and Thr2073 $NCBD_{D/P}^{ML}$. The corresponding tilt angle between $NCBD_{D/P}^{ML}$ and $NCBD_{D/P}^{AltAll}$ was estimated using Cα of Gln2068 $NCBD_{D/P}^{ML}$ and Leu2067 $NCBD_{D/P}^{AltAll}$, and Gln2072 $NCBD_{D/P}^{ML}$. For the third helix α3 (ML and human NCBD): Gln2106 $NCBD_{D/P}^{ML}$ and Ala2106 $NCBD_{Human}$, and Ala2099 $NCBD_{D/P}^{ML}$. Finally, α3 for ML and AltAll NCBD: Arg2104 $NCBD_{D/P}^{AltAll}$, Arg2104 $NCBD_{D/P}^{ML}$, and Phe2100 $NCBD_{D/P}^{AltAll}$. Additionally, the distance between the Cα of Ser2076 between $NCBD_{Human}$ and $NCBD_{D/P}^{ML}$ is 6.9 Å, while the corresponding distance between the $NCBD_{D/P}^{ML}$ and $NCBD_{D/P}^{AltAll}$ is 9.7 Å in the structural alignments. Schrödinger, L. & DeLano, W., 2020. *PyMOL*, Available at: http://www.pymol.org/pymol.

[2]Bernadó, P., Blackledge, M., and Sancho, J. (2006). Sequence-specific solvent accessibilities of protein residues in unfolded protein ensembles. *Biophysical Journal*, 91, 4536–4543. URL http://dx.doi.org/10.1529/biophysj.106.087528.
Estrada, J., Bernadó, P., Blackledge, M., and Sancho, J. (2009). ProtSA: a web application for calculating sequence specific protein solvent accessibilities in the unfolded ensemble. BMC *Bioinformatics*, 10, 104. URL http://dx.doi.org/10.1186/1471-2105-10-104.

Table S2. Summary of thermodynamic and kinetic paramaters of NCBD variants. The solutions were either 50 mM potassium formate buffer (pH 3.0), 150 mM NaCl or 20 mM sodium phosphate buffer (pH 7.4), 150 mM NaCl. The urea denaturation experiments were conducted at 277 K and the structural transition was monitored with CD at 222 nm. The data were fitted to a two-state model in GraphPad Prism. The parameters for the signal of the native state, the denatured and native baseline slopes, and the $m_{D\text{-}N}$ value were shared among the data sets in the curve fitting to allow fitting of the low stability $NCBD_{D/P}^{ML}$ and $NCBD_{D/P}^{AltAll}$ variants. The shared $m_{D\text{-}N}$ value was fitted as 0.82 ± 0.025 kcal mol^{-1}M^{-1}. The errors are the standard error from the curve fitting and likely a low estimate of the real error. The error for $\Delta G_{D\text{-}N}$ is the propagated fitting errors of $m_{D\text{-}N}$ and [Urea]$_{50\%}$. The temperature jump experiments were performed using 200 µM protein and the jump in temperature was from 277 to 285.5 K. The errors are the standard error from the curve fitting of an exponential function to kinetic traces (fluorescence versus time). Each kinetic trace contains 1000 individual data points.

NCBD variant	pH value	$\Delta G_{D\text{-}N}$ (kcal mol^{-1})	[Urea]$_{50\%}$ (M)	k_{obs} (s^{-1}) (temperature jump)	Amplitude (temperature jump)
$NCBD_{D/P}^{ML}$	3.0	0.38 ± 0.04	0.31 ± 0.03		
$NCBD_{D/P}^{ML}$	7.4	0.68 ± 0.04	0.55 ± 0.04		
$NCBD_{Human}$	3.0	1.61 ± 0.04	1.31 ± 0.05		
$NCBD_{Human}$	7.4	1.94 ± 0.05	1.58 ± 0.06		
$NCBD_{D/P}^{AltAll}$	3.0	0.22 ± 0.04	0.18 ± 0.03		
$NCBD_{Human}^{T2073W}$	3.0	3.24 ± 0.07	2.64 ± 0.10		
$NCBD_{Human}^{T2073W}$	7.4	3.46 ± 0.08	2.82 ± 0.11		
$NCBD_{D/P}^{T2073W}$	3.0	2.13 ± 0.04	1.73 ± 0.06		
$NCBD_{D/P}^{T2073W}$	7.4	2.08 ± 0.04	1.70 ± 0.06	2400 ± 200	1.06 ± 0.06
$NCBD_{D/P}^{L2067W}$	7.4			1530 ± 50	0.91 ± 0.02
$NCBD_{D/P}^{S2078W}$	7.4			700 ± 60	0.35 ± 0.01
$NCBD_{D/P}^{H2107W}$	7.4			820 ± 80	0.47 ± 0.02
$NCBD_{D/P}^{Q2108W}$	7.4			960 ± 60	0.73 ± 0.02
