# Supplementary material for: The dynamic properties of a nuclear coactivator binding domain are evolutionarily conserved
Source: Commun Biol. 2022 Mar 30;5:286. doi: 10.1038/s42003-022-03217-y (PMC8967867; doi:10.1038/s42003-022-03217-y)
Supplement: Supplementary file 4 — Reporting Summary [file 42003_2022_3217_MOESM4_ESM.pdf]

nature portfolio

Corresponding author(s): *Double-blind peer review submissions: write DBPR and your manuscript number here instead of author names.*

Last updated by author(s): *YYYY-MM-DD*

Reporting Summary

Nature Portfolio wishes to improve the reproducibility of the work that we publish. This form provides structure for consistency and transparency in reporting. For further information on Nature Portfolio policies, see our Editorial Policies and the Editorial Policy Checklist.

Statistics

For all statistical analyses, confirm that the following items are present in the figure legend, table legend, main text, or Methods section.

n/a	Confirmed	
☐	☒	The exact sample size (n) for each experimental group/condition, given as a discrete number and unit of measurement
☒	☐	A statement on whether measurements were taken from distinct samples or whether the same sample was measured repeatedly
☒	☐	The statistical test(s) used AND whether they are one- or two-sided<br>*Only common tests should be described solely by name; describe more complex techniques in the Methods section.*
☒	☐	A description of all covariates tested
☒	☐	A description of any assumptions or corrections, such as tests of normality and adjustment for multiple comparisons
☐	☒	A full description of the statistical parameters including central tendency (e.g. means) or other basic estimates (e.g. regression coefficient) AND variation (e.g. standard deviation) or associated estimates of uncertainty (e.g. confidence intervals)
☒	☐	For null hypothesis testing, the test statistic (e.g. F, t, r) with confidence intervals, effect sizes, degrees of freedom and P value noted<br>*Give P values as exact values whenever suitable.*
☒	☐	For Bayesian analysis, information on the choice of priors and Markov chain Monte Carlo settings
☒	☐	For hierarchical and complex designs, identification of the appropriate level for tests and full reporting of outcomes
☒	☐	Estimates of effect sizes (e.g. Cohen's d, Pearson's r), indicating how they were calculated

Our web collection on statistics for biologists contains articles on many of the points above.

Software and code

Policy information about availability of computer code

Data collection	Commercial softwares provided with the instruments were used for data collection and data analysis for circular dichroism, stopped flow spectroscopy and isothermal titration calorimetry experiments. Bruker Topspin was used for NMR data
Data analysis	GraphPad Prism version 9.3.1 was used together with the commercial softwares for for data analysis of circular dichroism, stopped flow spectroscopy and isothermal titration calorimetry experiments. CYANA 3.93 was used for structure calculation, nmrpipe for NMR processing, and ccpnmr for NMR assignment

For manuscripts utilizing custom algorithms or software that are central to the research but not yet described in published literature, software must be made available to editors and reviewers. We strongly encourage code deposition in a community repository (e.g. GitHub). See the Nature Portfolio guidelines for submitting code & software for further information.

Data

Policy information about availability of data

All manuscripts must include a data availability statement. This statement should provide the following information, where applicable:

- Accession codes, unique identifiers, or web links for publicly available datasets
- A description of any restrictions on data availability
- For clinical datasets or third party data, please ensure that the statement adheres to our policy

All data and clones are available upon request. Assignments and structural coordinates have been deposited in the RCSB protein data bank with pdb code: 7OSR (accession number BMRB ID 34635) (NCBDD/PML) and 7OSW (accession number BMRB ID 34636) (NCBDD/PAltAll). All other data are provided in the paper.

Field-specific reporting

Please select the one below that is the best fit for your research. If you are not sure, read the appropriate sections before making your selection.

☒ Life sciences ☐ Behavioural & social sciences ☐ Ecological, evolutionary & environmental sciences

For a reference copy of the document with all sections, see nature.com/documents/nr-reporting-summary-flat.pdf

Life sciences study design

All studies must disclose on these points even when the disclosure is negative.

Sample size	Sample sizes were typical of the kind of biophysical experiments reported. While individual data points were not replicated for urea and temperature denaturation experiments, the large number of data points serve as internal controls. Kinetic transients were averages of several individual acquisitions, each containing 200-1000 data points.
Data exclusions	No data were excluded
Replication	All data are presented in the main text or in supplemental information. Some of the experiments (stopped flow, circular dichroism) are very accurate and precise and more data points in the respective data set was favored over replication. For example, while individual data points were not replicated for urea and temperature denaturation experiments, the large number of data points serve as internal controls. Kinetic transients were averages of several individual acquisitions, each containing 200-1000 data points.
Randomization	Randomization is usually not used in the type of biophysical experiments presented in this paper, since both accuracy and precision are very high.
Blinding	Blinding is usually not used in the type of biophysical experiments presented in this paper, since both accuracy and precision are very high.

Reporting for specific materials, systems and methods

We require information from authors about some types of materials, experimental systems and methods used in many studies. Here, indicate whether each material, system or method listed is relevant to your study. If you are not sure if a list item applies to your research, read the appropriate section before selecting a response.

Materials & experimental systems

n/a	Involved in the study	
☒	☐	Antibodies
☒	☐	Eukaryotic cell lines
☒	☐	Palaeontology and archaeology
☒	☐	Animals and other organisms
☒	☐	Human research participants
☒	☐	Clinical data
☒	☐	Dual use research of concern

Methods

n/a	Involved in the study	
☒	☐	ChIP-seq
☒	☐	Flow cytometry
☒	☐	MRI-based neuroimaging
